# Density-Dependent Changes in Root Exudates, Rhizosphere Microbes, and Soil Metabolites of *Solanum rostratum*

**DOI:** 10.3390/plants15213439

**Published:** 2026-11-05

**Authors:** Yaxing Wu, Fang Wang, Shuai Liu, Tiancheng Dai, Jing Deng, Lamei Jiang, Amanula Yimingniyazi, Shanshan Wang

**Affiliations:** 1 College of Resources and Environment, Xinjiang Agricultural University, Ürümqi 830052, China; 15378842955@163.com; 2 Xinjiang Key Laboratory for Ecological Adaptation and Evolution of Extreme Environment Organisms, College of Life Sciences, Xinjiang Agricultural University, Ürümqi 830052, China13568113613@163.com (J.D.);

**Keywords:** intraspecific competition, root exudates, rhizosphere microbiome, soil metabolomics, multi-omics integration, *Solanum rostratum*

## Abstract

Intraspecific competition shapes high-density monodominant communities of invasive plants, but how density is associated with rhizosphere microbial reassembly and soil metabolite accumulation via root exudates remains unclear. We established four planting densities (1, 3, 5, and 10 plants per pot) and integrated growth and photosynthetic physiology, root exudate metabolomics, rhizosphere metagenomics, and soil metabolomics using Mantel tests and variable-level correlation analysis. Increasing density reduced all traits except plant height, which showed a slight, non-significant increase followed by a significant decrease. Root exudates shifted from phenolic acid/terpenoid dominance at low density to sugar/peptide dominance at high density. Low-density-enriched bacterial genera were positively correlated with biomass, whereas high-density-enriched genera (e.g., *Ilumatobacter*, *Allosphingosinicella*) were negatively correlated. Soil metabolites such as Glutarate Semialdehyde and Sutherlandin D were negatively correlated with biomass, root traits, and photosynthesis. Mantel tests showed strong correlations between plant phenotypes and root exudates (*r* = 0.80) and between exudates and soil metabolites (*r* = 0.83). Variable-level analysis linked phenolic acid exudates to putatively growth-associated bacteria (e.g., *Flavitalea*, *Blastococcus*) and sugar/peptide exudates to putatively stress-adapted bacteria (e.g., *Allosphingosinicella*, *Ilumatobacter*), which were positively correlated with Glutarate Semialdehyde and Sutherlandin D accumulation. These results indicate that density-associated exudate changes synchronized with rhizosphere microbial reassembly and soil metabolite accumulation, forming an “exudate–microbe–metabolite” cascade. This cascade may buffer against high-density intraspecific competition and help explain monodominant community formation in invaded ranges.

## 1. Introduction

Plant density profoundly affects plant morphogenesis, physiological traits, and rhizosphere processes by altering resource competition patterns. In invasion ecology, many invasive plants often form high-density monodominant communities; therefore, intraspecific competition may serve as an important regulatory force for their adaptive expansion. For example, *Mikania micrantha* exhibits density-dependent effects such as increased reproductive allocation and altered dispersal traits under high density [1]. After invasion, *Solanum rostratum* also shows contiguous high-density growth (Supplementary Figure S1); however, how intraspecific competition systematically regulates its rhizosphere processes remains unclear. Recent studies have shown that intraspecific competition can alter root exudate metabolic profiles [2], and plant density can reshape rhizosphere chemistry [3]. Density-associated exudate remodeling may also exacerbate negative plant–soil feedback [4]. In addition, rhizosphere microbes can influence intraspecific competition in invasive plants [5], and invasive plant competitiveness may be jointly regulated by nitrogen use strategies and the rhizosphere microbiome [6].

Root exudates are central chemical mediators in plant–soil microbial interactions. Plants can actively recruit beneficial microbes and interfere with nutrient cycling through root exudates, thereby playing a key role in plant invasion. Invasive plants can suppress native plants or strengthen mutualistic symbiosis through specific secondary metabolites such as (±)-catechin and quercetin [7,8,9]; for instance, *Mikania micrantha* can reshape rhizosphere nitrogen cycling through enzyme activation, microbial recruitment, and allelopathic regulation, thereby gaining a competitive advantage [10]. Current rhizosphere studies on invasive plants have mostly focused on interspecific competition; however, whether root exudate composition changes when intraspecific competition intensifies and further associates with rhizosphere bacterial community reassembly and soil metabolite accumulation remains poorly studied.

*Solanum rostratum* is a noxious annual invasive plant native to North America that has rapidly spread across many regions of China, posing a serious threat to agriculture and ecosystems. Existing studies have mostly focused on its seed traits, allelopathic and autotoxic effects [11,12], whereas how intraspecific competition regulates the multilevel interactions among its seedling growth, physiology, and rhizosphere microecosystem still requires further investigation. Given the ecological reality that this species often forms high-density monodominant communities in invaded areas, revealing the rhizosphere response mechanisms under different densities is of great significance for understanding its invasion mechanisms and formulating control strategies. Intraspecific variation in indirect plant–soil feedbacks can also influence invasion success [13].

In this study, we established four plant density treatments (1, 3, 5, and 10 plants per pot) to systematically investigate the effects of intraspecific competition on the seedling morphology, photosynthetic physiology, root exudates, rhizosphere bacterial community, and soil metabolites of *S. rostratum*. We aimed to elucidate the response pattern of the “exudate–microbe–metabolite” cascade under intraspecific competition, thereby providing a theoretical basis for the ecological control of invasive plants. We hypothesized that intensified intraspecific competition would alter root exudate composition, be associated with a shift in the rhizosphere bacterial community from putatively growth-associated taxa to putatively stress-adapted taxa, and be accompanied by the accumulation of soil metabolites related to growth decline, forming potential rhizosphere feedback.

## 2. Results

### 2.1. Plant Density Profoundly Suppressed Seedling Growth and Induced Stomatal Limitation of Photosynthesis

Root system architecture exhibited the most striking response to plant density: root scans revealed a progressive simplification of the root network from a densely branched, well-developed system in the One-plant group to a sparse, attenuated structure in the Ten-plant group (Figure S2). Quantitative analysis confirmed that with the increase in density (from One to Ten plants per pot), total root length declined by 91.8% (from 446.25 to 36.79 cm), total root surface area by 91.2% (from 40.83 to 3.61 cm^2^), total root volume by 94.1% (from 0.974 to 0.057 cm^3^), and the number of root tips by 93.2% (from 893 to 61; Supplementary Table S1).

**Figure 1:**
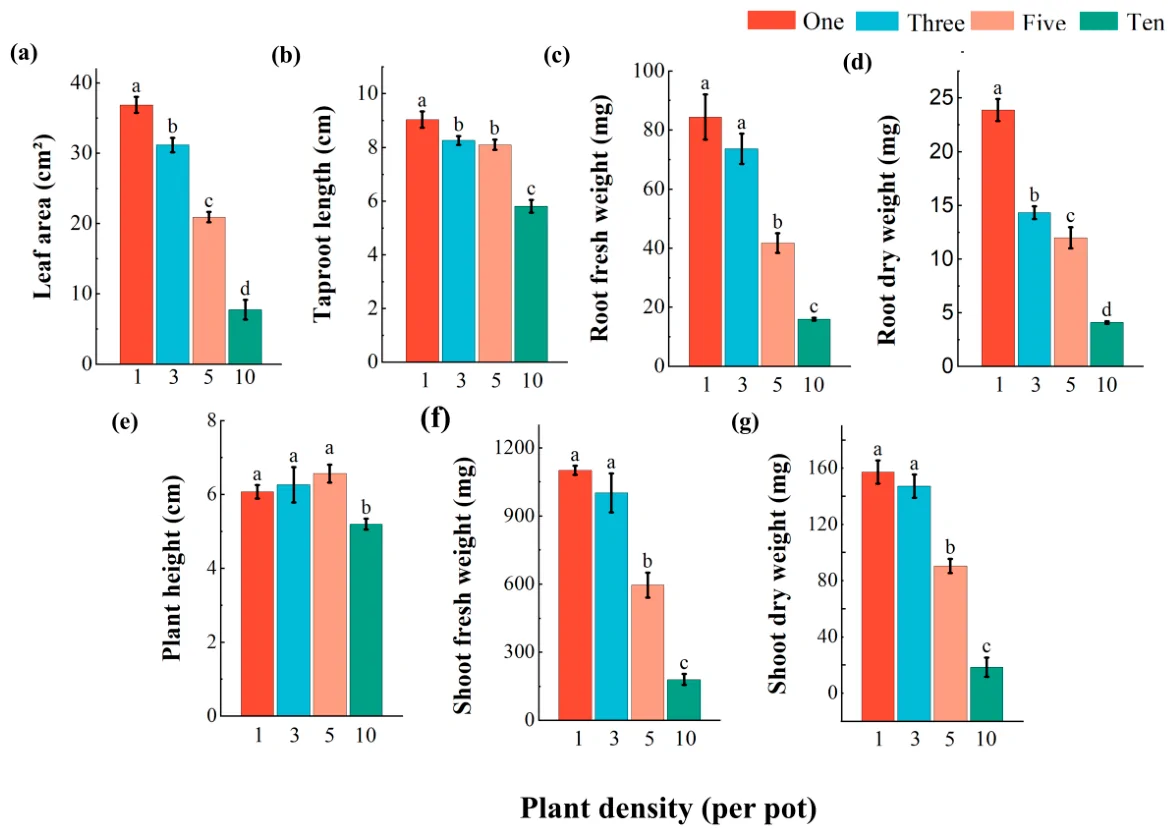
Effects of different plant density on seedling growth parameters of *S. rostratum*. (**a**) Leaf area. (**b**) Taproot length. (**c**) Root fresh weight. (**d**) Root dry weight. (**e**) Plant height. (**f**) Shoot fresh weight. (**g**) Shoot dry weight. Data are presented as means ± standard deviations (*n* = 3). Different lowercase letters indicate significant differences among plant densities (*p* < 0.05).

Consistently, all aboveground and belowground biomass parameters declined significantly with the increase in density (*p* < 0.05; Figure 1). Compared with the One group, the Ten group showed reductions of 78.94%, 81.05%, 82.84%, 83.56%, and 78.25% in leaf area, root fresh weight, root dry weight, shoot fresh weight, and shoot dry weight, respectively (Figure 1a,c,d,f,g). Taproot length also decreased markedly by 35.66% (Figure 1b). Notably, plant height followed a different trajectory, exhibiting a slight, non-significant increase from the One to the Five group (+8.24%, *p* > 0.05) before declining significantly at the highest density (−14.33% in Ten vs. One, *p* < 0.05; Figure 1e). Effect size analysis (Cohen’s *d*, One vs. Ten) corroborated these patterns, revealing very large density effects across all traits (|*d*| > 3.7). Plant height exhibited a comparatively smaller effect (*d* = 5.26). Biomass traits and some gas exchange parameters (*E* and *G*_s_) were affected more severely (|*d*| = 12.6–41.7), whereas *A* and *C*_i_ showed smaller but still large effects (*d* = 3.76 and 6.04, respectively; Supplementary Table S2).

**Figure 2:**
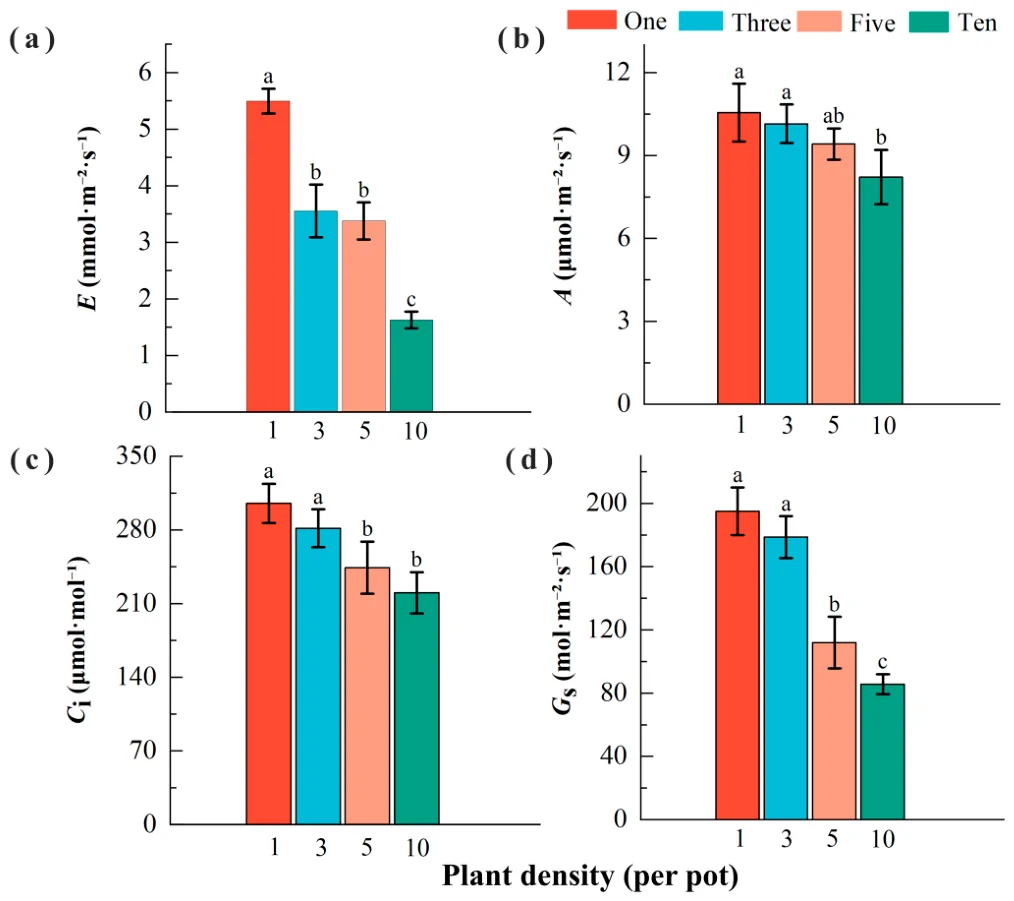
Effects of different plant densities on seedling physiological parameters of *S. rostratum*. (**a**) Transpiration rate *(E*). (**b**) Net photosynthetic rate (*A*). (**c**) Intercellular CO_2_ concentration (*C*_i_). (**d**) Stomatal conductance (*G*_s_). Data are presented as means ± standard deviations (*n* = 3). Different lowercase letters indicate significant differences among plant densities (*p* < 0.05).

The photosynthetic performance of seedlings was equally sensitive to density (Figure 2). The net photosynthetic rate (*A*) declined progressively with the increase in density, with the Ten group showing a significant reduction relative to the One group (Figure 2b). This decline in A was accompanied by concurrent decreases in both stomatal conductance (*G*_s_; Figure 2d) and intercellular CO_2_ concentration (*C*_i_; Figure 2c), while the transpiration rate (*E*) dropped by over 70% in the Ten group (Figure 2a). The synchronous reductions in *A*, *G*_s_, and *C*_i_ indicate that the suppression of photosynthesis under high-density conditions was primarily attributable to stomatal limitation rather than non-stomatal constraints. Collectively, these results demonstrate that intraspecific competition under high density imposes severe carbon assimilation deficits on *S. rostratum* seedlings, which cascade into substantial reductions in biomass accumulation and root system development.

### 2.2. Density-Associated Reprogramming of Root Exudate Profiles

Plant density triggered a systematic reprogramming of the root exudate metabolome of *S. rostratum*. Partial least squares-discriminant analysis (PLS-DA) revealed a clear and progressive separation of samples along the density gradient, with all four treatments occupying distinct ordination spaces (*R*^2^ > 0.99, *p* = 0.001; Supplementary Figure S3a). Pairwise comparison revealed that 419 metabolites were significantly up-regulated and 101 were down-regulated in the Ten group relative to the One group (Supplementary Figure S3b), suggesting that high-density competition triggered the release of a large suite of stress-associated metabolites.

**Figure 3:**
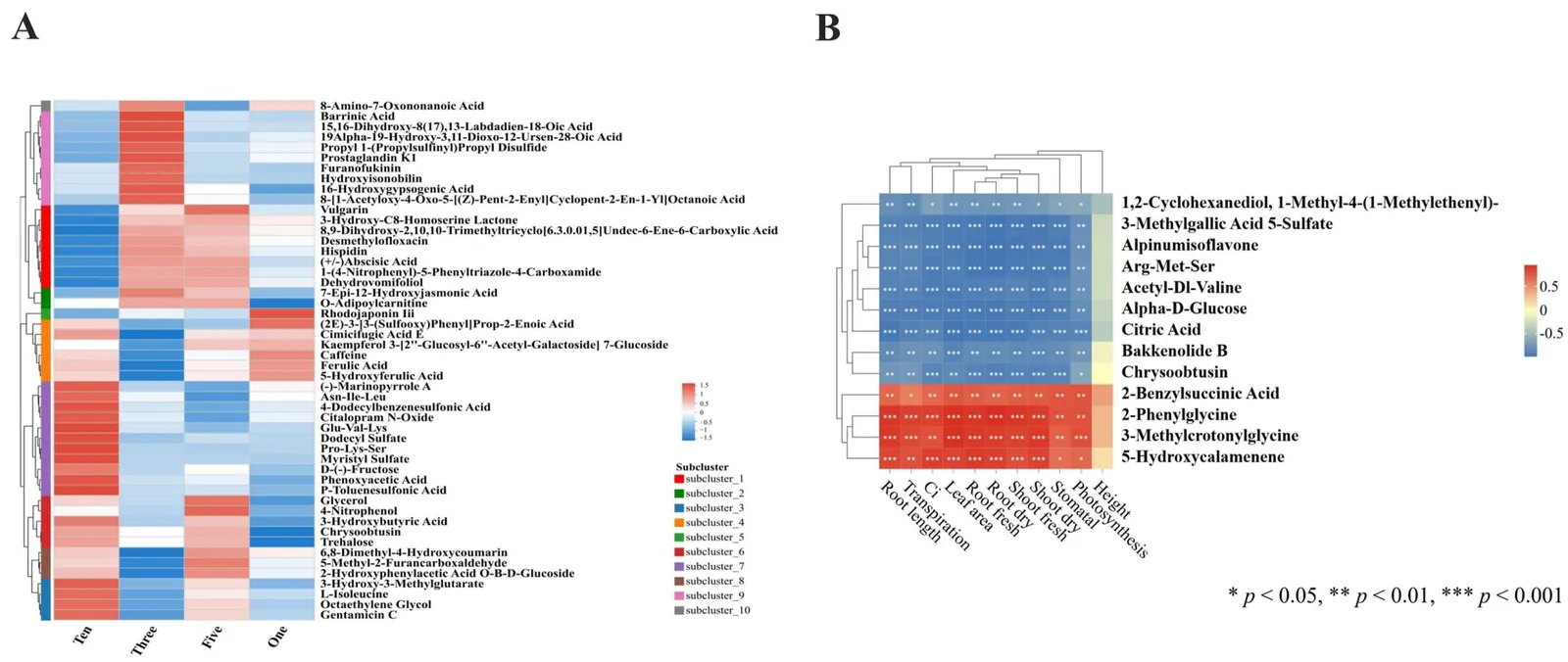
Differential profiles of root exudates from *S. rostratum* and their correlation with seedling growth parameters. (**A**) Clustering heatmap of root exudates showing the abundance trends with increase in planting density (One, Three, Five, and Ten groups; red, high abundance; blue, low abundance). (**B**) Spearman correlation heatmap of 13 significantly correlated plant-derived metabolites and seedling growth and physiological parameters. Red indicates a positive correlation; blue indicates a negative correlation. One, Three, Five, and Ten represent 1, 3, 5, and 10 plants per pot, respectively. * *p* < 0.05, ** *p* < 0.01, *** *p* < 0.001.

The hierarchical clustering of root exudates revealed clear density-dependent shifts in the exudation profile of *S. rostratum* (Figure 3A). Five and One clustered together first, followed by Three, whereas Ten formed a distinct branch, indicating that high planting density caused the strongest divergence in exudate composition. The One group was characterized by Rhodojaponin III, Caffeine, and Ferulic Acid; the Three group by Barrinic Acid, 19Alpha-19-Hydroxy-3,11-Dioxo-12-Ursen-28-Oic Acid, and 16-Hydroxygypsogenic acid; the Five group by 4-Nitrophenol, Vulgarin, and Glycerol; and the Ten group by Pro-Lys-Ser, P-Toluenesulfonic Acid, and Phenoxyacetic acid.

To identify core metabolites responsive to density across the gradient, we applied an intersection-based screening strategy using volcano plots and Venn diagrams (Supplementary Figure S4). A total of 955 metabolites were consistently differentially abundant across the three pairwise comparisons. Among these, only 13 metabolites could be putatively annotated against the HMDB and Metlin databases. The relatively low annotation rate is a common limitation of non-targeted metabolomics for complex plant–soil systems, primarily due to the lack of authentic standards for uncharacterized secondary metabolites. However, it should be noted that the core metabolites identified in this study exhibited high mass accuracy (<10 ppm) and were assigned to B(i)/B(ii) identification levels based on MS/MS fragmentation matching. Therefore, we retained these 13 putatively annotated metabolites for downstream correlation analysis (Figure 3B). Spearman correlation analysis revealed two contrasting response patterns. Four metabolites—2-Benzylsuccinic Acid, 2-Phenylglycine, 3-Methylcrotonylglycine, and 5-Hydroxycalamenene—exhibited significant positive correlations with all measured growth and physiological traits, including leaf area, taproot length, root and shoot biomass, *A*, *E*, *G*_s_, and *C*_i_ (*p* < 0.05). Conversely, the remaining nine metabolites (1,2-Cyclohexanediol, 1-Methyl-4-(1-Methylethenyl)-; 3-Methylgallic Acid 5-Sulfate; Acetyl-DL-Valine; Alpha-D-Glucose; Alpinumisoflavone; Arg-Met-Ser; Bakkenolide B; Chrysoobtusin; and Citric Acid) showed significant negative correlations with these traits.

### 2.3. Intraspecific Competition Restructured Rhizosphere Microbial Communities and Exudate–Microbe Associations

**Figure 4:**
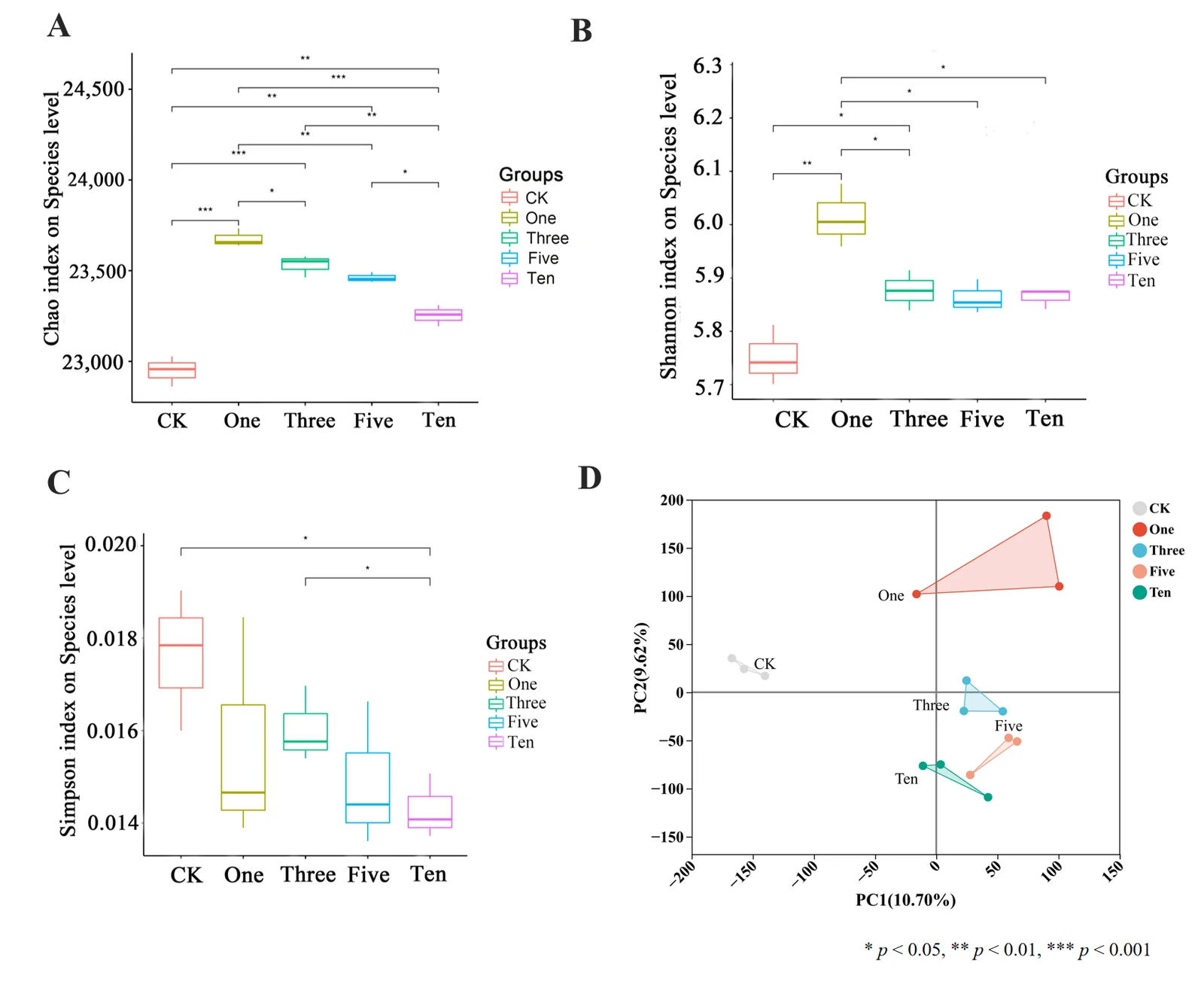
Diversity of rhizosphere soil microbial communities of *S. rostratum* under different plant densities. (**A**) Chao index. (**B**) Shannon index. (**C**) Simpson index. (**D**) Principal component analysis (PCA) based on Bray–Curtis distance. CK: no-plant control; One, Three, Five, and Ten represent 1, 3, 5, and 10 plants per pot, respectively (*n* = 3). In the box plots (**A**–**C**), the horizontal line within the box indicates the median, and the box edges represent the interquartile range (IQR). Asterisks (*) indicate significant differences (Tukey–Kramer test, * *p* < 0.05, ** *p* < 0.01, *** *p* < 0.001).

Plant density profoundly reshaped both the diversity and assembly of rhizosphere microbial communities of *S. rostratum*. Species annotation analysis based on a non-redundant gene set revealed the shared and unique taxa across treatment groups (Figure S5). The treatments shared 4241 core taxa, and the UpSet plot illustrated the shared and unique characteristics among different group combinations. α-Diversity analyses revealed that the introduction of *S. rostratum* consistently enriched bacterial diversity relative to the unplanted control: both the Chao and Shannon indices were significantly elevated across all planted treatments, while the Simpson index was correspondingly reduced (Figure 4A–C). Notably, both richness (Chao) and evenness (Shannon) peaked in the One group and declined progressively with the increase in density, although they remained higher than CK even in the Ten group. The Simpson index showed an overall declining trend with the increase in density, reaching its minimum in the Ten group. Compared with the unplanted control (CK), the Simpson index was significantly lower in the Five and Ten groups (*p* < 0.05; Figure 4C), whereas the One and Three groups did not differ significantly from CK. Bray–Curtis-based PCA (Figure 4D) revealed notable separation between the no-plant control (CK) and most planted treatments along the PC1 axis, indicating a strong rhizosphere effect of *S. rostratum* on soil bacterial community composition. Notably, the One treatment exhibited pronounced separation from all other treatments along the PC2 axis. In contrast, the planted treatments exhibited density-dependent clustering, with the higher-density treatments (Three, Five, and Ten) showing a progressive shift in community structure along the PC1 axis. These results suggest that intraspecific crowding continuously reshapes the rhizosphere microbiome.

**Figure 5:**
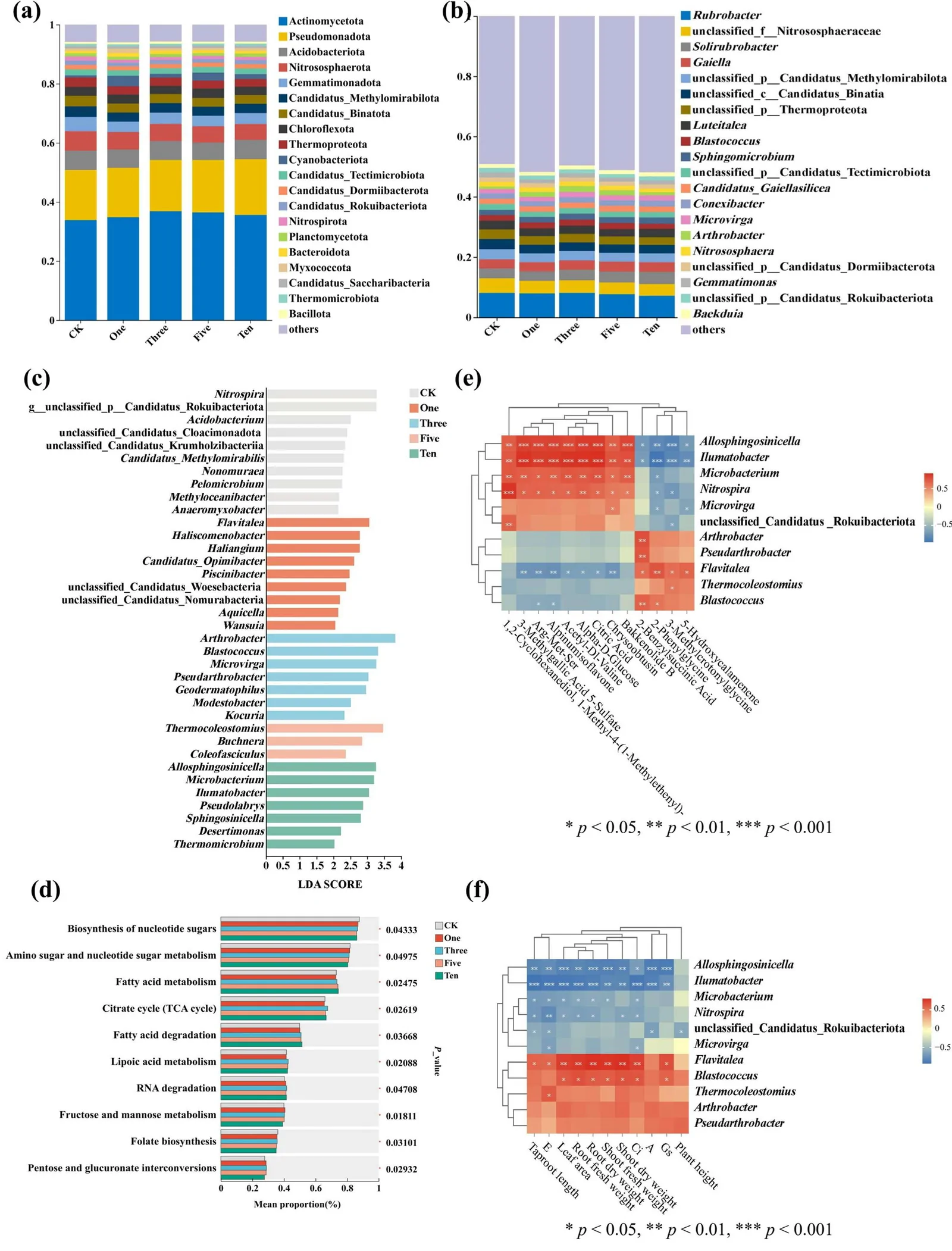
Rhizosphere soil microbial community structure, differentially abundant taxa, and their correlations with plant growth and physiological parameters of *S. rostratum* under different plant densities. (**a**) Relative abundance bar chart at the phylum level. (**b**) Relative abundance bar chart at the genus level. (**c**) LDA score bar chart from LEfSe analysis (LDA > 3, *p* < 0.05). (**d**) Results of the one-way ANOVA followed by Tukey–Kramer post-hoc test comparing dominant rhizosphere genera among different plant density treatments. (**e**) Spearman correlation heatmap of differentially abundant bacterial genera and root exudates. Data are presented as exploratory correlation analyses based on *n* = 3. (**f**) Spearman correlation heatmap of differentially abundant bacterial genera and plant growth and physiological parameters. Data are presented as exploratory correlation analyses based on *n* = 3. CK: no-plant control; 1, 3, 5, 10 represent 1, 3, 5, and 10 plants per pot, respectively. In (**a**,**b**), data are presented as means ± standard deviations, and different lowercase letters indicate significant differences among treatments (*p* < 0.05). In (**d**), one-way ANOVA followed by Tukey–Kramer post-hoc test was performed on genus-level TPM abundance, with FDR correction for multiple testing (*p* < 0.05).Asterisks indicate significant differences (* *p* < 0.05, ** *p* < 0.01, *** *p* < 0.001).

At the phylum level, Actinomycetota, Pseudomonadota, and Acidobacteriota dominated all rhizosphere communities (Figure 5a). Pseudomonadota, a phylum often enriched under stress and disturbance, was markedly more abundant in the Ten group than in the One group. This suggests a density-associated shift toward copiotrophic and potentially stress-adapted taxa. At the genus level (Figure 5b), *Rubrobacter* maintained consistently high abundance across treatments, whereas *Nitrososphaera* showed no significant differences across the density gradient.

LEfSe analysis (LDA > 3, *p* < 0.05) identified treatment-specific biomarker taxa (Figure 5c): *Nitrospira* characterized the CK group; *Flavitalea* the One group; *Arthrobacter* the Three group; *Thermocoleostomius* the Five group; and *Allosphingosinicella* together with *Microbacterium* marked the Ten group. One-way ANOVA followed by Tukey–Kramer post-hoc tests on dominant genera further resolved their density–response trajectories (Figure 5d): *Blastococcus* and *Arthrobacter* peaked at intermediate densities (Three group) before declining; *Allosphingosinicella*, *Ilumatobacter*, and *Chloracidobacterium* increased monotonically, peaking in the Ten group; *Nitrososphaera* declined gradually with density, reaching its minimum in the Ten group; *Nitrospira* dropped sharply upon plant introduction (One group) before partially recovering at higher densities, whereas *Microvirga* and *Geodermatophilus* exhibited only moderate fluctuations. In total, 11 genera showed significant density-dependent differentiation (LDA > 3, *p* < 0.05; Supplementary Table S3).

Spearman correlation analysis revealed that these density-responsive genera assembled into two functionally contrasting modules linked to root exudates (Figure 5e). *Allosphingosinicella*, *Ilumatobacter*, *Microbacterium*, and *Nitrospira* were positively correlated with stress-associated metabolites (e.g., 1,2-Cyclohexanediol, 1-Methyl-4-(1-Methylethenyl)-, Citric Acid, Chrysoobtusin) and negatively correlated with putatively growth-associated phenolic acids (e.g., 2-Benzylsuccinic Acid, 5-Hydroxycalamenene), whereas *Flavitalea* exhibited the opposite pattern. Several genera displayed more specific exudate affinities: *Arthrobacter* and *Pseudarthrobacter* correlated solely with 2-Benzylsuccinic Acid; *Thermocoleostomius* with 3-Methylcrotonylglycine; *Blastococcus* was positively associated with 2-Benzylsuccinic Acid and 2-Phenylglycine but negatively with Arg-Met-Ser and Alpinumisoflavone; unclassified_Candidatus_Rokuibacteriota was positively associated with 1,2-Cyclohexanediol but negatively with 3-Methylcrotonylglycine; and *Microvirga* showed positive correlations with Chrysoobtusin but negative correlations with 2-Phenylglycine and 5-Hydroxycalamenene.

These exudate-defined microbial modules translated directly into divergent associations with plant performance (Figure 5f). The potentially stress-adapted module (*Allosphingosinicella*, *Ilumatobacter*, *Microbacterium*, and *Nitrospira*) was broadly and negatively correlated with biomass and gas exchange parameters, with Ilumatobacter exhibiting significant negative correlations with all measured traits except plant height. Conversely, the putatively growth-associated module (*Flavitalea* and *Blastococcus*) was positively correlated with most growth and physiological traits. Notably, unclassified_Candidatus_Rokuibacteriota was the only genus significantly negatively correlated with plant height, while *Thermocoleostomius* correlated positively only with *E*. No significant correlations were detected for *Arthrobacter* or *Pseudarthrobacter.* Collectively, these results suggest that intraspecific competition was associated with a coordinated shift in the rhizosphere community in which changes in root exudation co-occurred with the enrichment of putatively stress-adapted taxa and a decline in putatively growth-associated symbionts, potentially forming a chemically linked feedback loop between plant performance and microbial community assembly.

**Figure 6:**
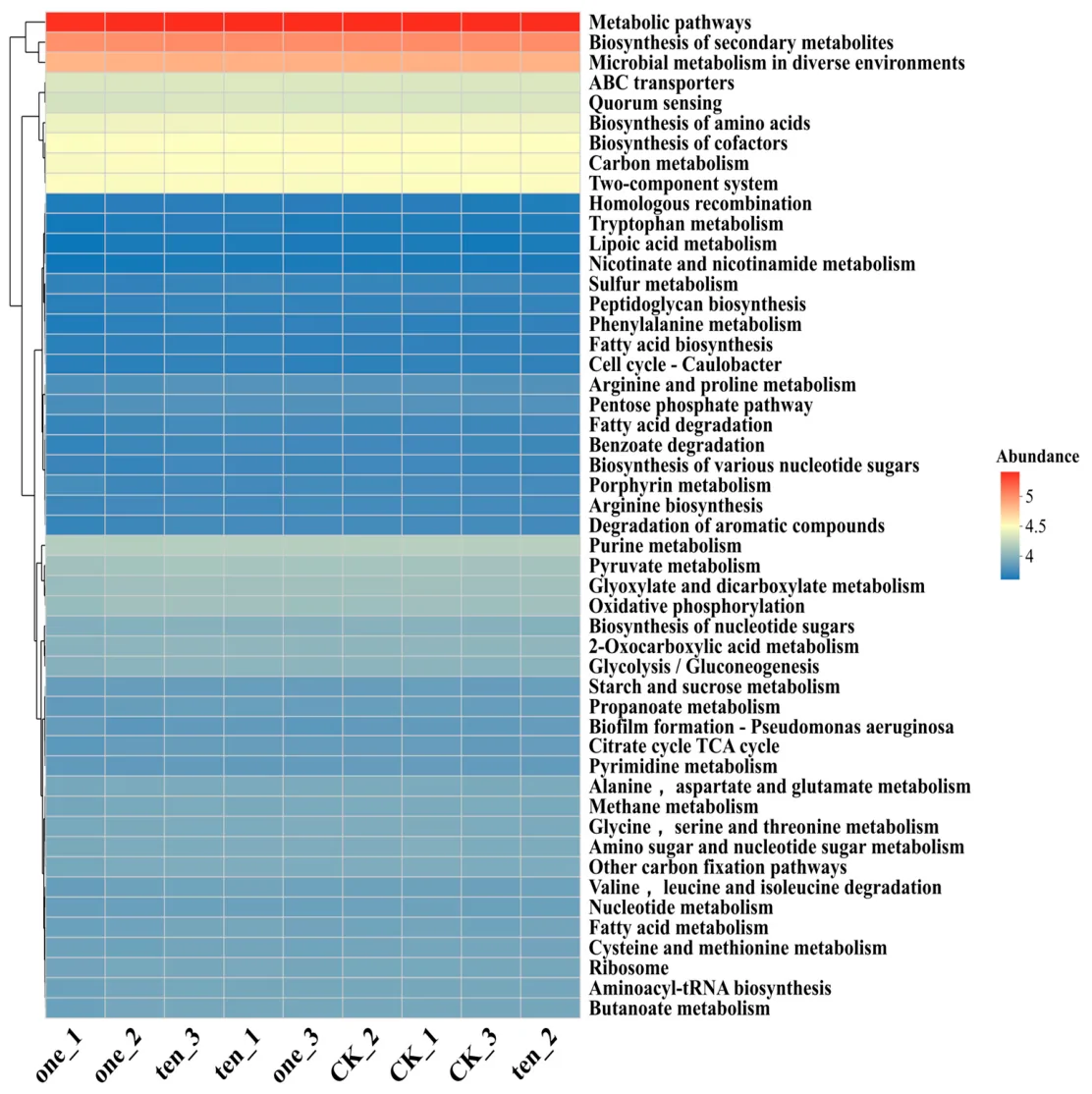
KEGG Pathway Level 3 functional heatmap of the rhizosphere microbiome. The heatmap shows the top 50 most abundant pathways across CK, One, and Ten treatments. The color gradient represents the relative abundance (log_10_-transformed TPM values), with red indicating higher abundance and blue indicating lower abundance. Pathways related to environmental sensing and transport (e.g., Two-component system and ABC transporters) show higher relative abundances in the high-density (Ten) group.

To further explore the functional potential of the rhizosphere microbiome, metagenomic functional profiles were analyzed. At KEGG Pathway Level 2, overall functional categories remained relatively stable across densities, suggesting high functional redundancy (Figure S6). However, at Pathway Level 3, pathways related to environmental sensing and transport (e.g., Two-component system and ABC transporters) were enriched under high density (Figure 6). CAZy analysis further revealed that core carbohydrate-active enzymes, such as Glycoside Hydrolases (GHs), remained stable (Figure S7). These functional shifts support the response of putatively stress-adapted taxa under intensified competition.

### 2.4. High-Density Competition Reshaped the Rhizosphere Metabolome and Enriched Metabolites Potentially Related to Growth Decline

The response of the rhizosphere soil metabolome to plant density mirrored the patterns observed for root exudates, indicating a tight chemical coupling between root secretion and the bulk soil chemical environment. PLS-DA revealed a clear treatment-dependent separation of soil metabolite profiles (*R* = 0.84, *p* = 0.001; Supplementary Figure S8a). Consistent with the homogenization pattern observed in microbial community structure (Figure 4D), the Three, Five, and Ten groups clustered tightly in ordination space, whereas the CK and One groups occupied distinct positions, suggesting that elevated intraspecific competition was associated with convergent shifts in soil chemistry. Pairwise comparison between the One and Ten groups identified 36 significantly up-regulated and 142 down-regulated metabolites (Supplementary Figure S8b).

**Figure 7:**
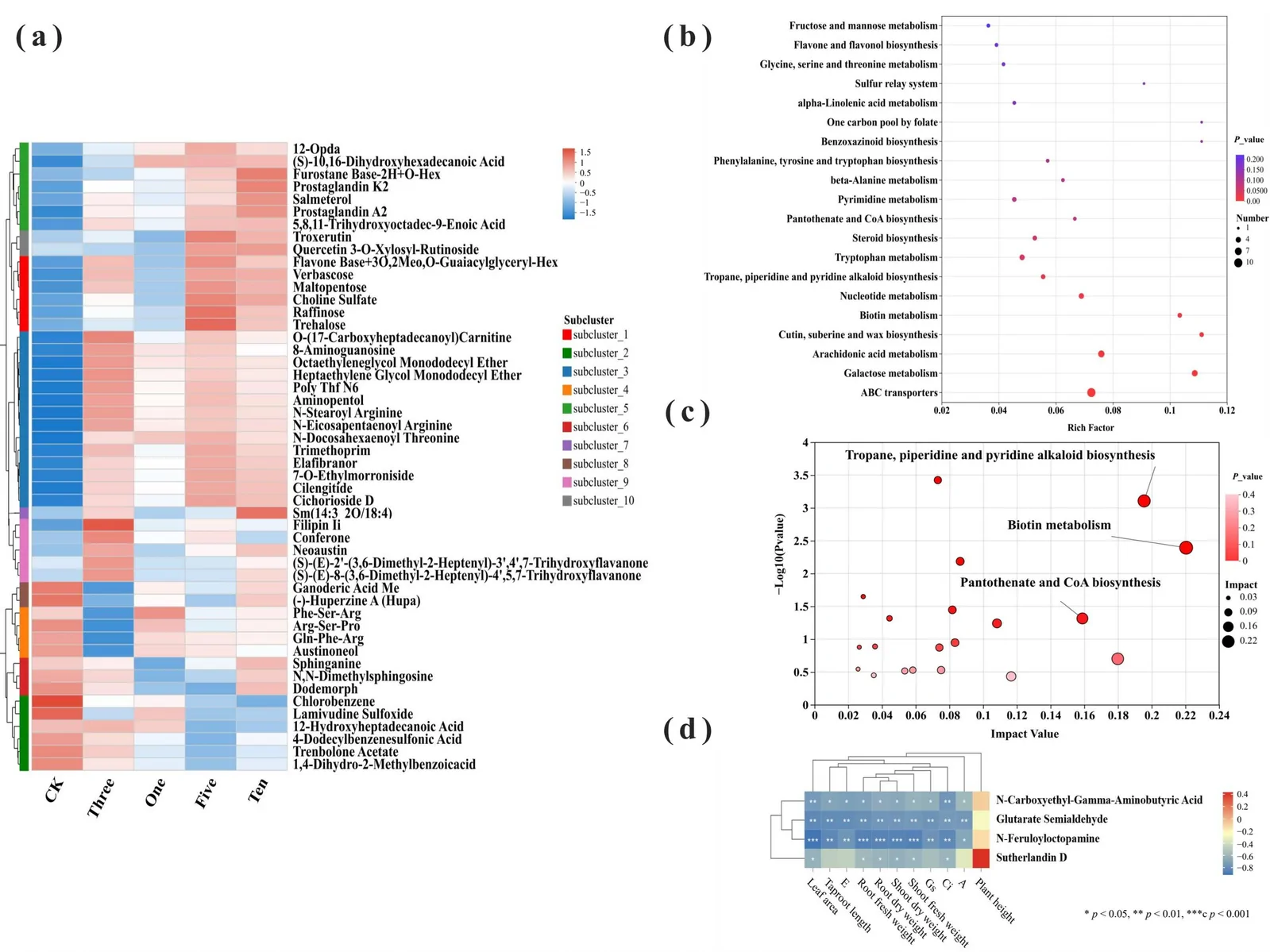
Differential rhizosphere soil metabolite profiles, KEGG pathway enrichment analysis, KEGG topological analysis, and their correlations with seedling growth of *S. rostratum* under different plant densities. (**a**) Clustering heatmap of soil metabolites showing abundance trends across the CK, 1-, 3-, 5-, and 10-plant treatment groups (red, high abundance; blue, low abundance). (**b**) KEGG pathway enrichment bubble plot. The horizontal axis represents the enrichment factor, and the vertical axis represents the pathway name. Bubble size indicates the number of differentially accumulated metabolites, and bubble color reflects enrichment significance (*p*-value). (**c**) KEGG topological analysis. The horizontal axis represents the pathway impact value, and the vertical axis represents −log_10_ (*p*-value). Bubble size indicates the pathway impact value, and bubble color reflects *p*-value significance. (**d**) Spearman correlation heatmap of key differentially accumulated soil metabolites and seedling growth parameters. Data in (**a**) are presented as means ± standard deviations. CK: no-plant control; 1, 3, 5, 10 represent 1, 3, 5, and 10 plants per pot, respectively. * *p* < 0.05, ** *p* < 0.01, *** *p* < 0.001.

The hierarchical clustering heatmap (Figure 7a) further resolved treatment-specific metabolite signatures. The unplanted CK group was dominated by aromatic hydrocarbons and pharmaceutical analogs (e.g., Chlorobenzene and Lamivudine Sulfoxide), reflecting baseline soil chemistry. Upon plant introduction, the One group exhibited a metabolome enriched in peptides and long-chain fatty acid derivatives (e.g., Phe-Ser-Arg and (S)-10,16-Dihydroxyhexadecanoic Acid). With the increase in density, the metabolite composition shifted progressively: the Three group was enriched in polyketide antibiotics and long-chain acylcarnitines (e.g., Filipin II and O-(17-Carboxyheptadecanoyl)carnitine); the Five group was dominated by osmoregulatory carbohydrates (e.g., Trehalose and Raffinose); and the Ten group was characterized by sphingolipids and steroid glycosides (e.g., SM(14:3_2O/18:4) and Furostane Base-2H+O-Hex). This progressive transition—from peptide- and fatty acid-dominated profiles to osmolyte- and membrane-lipid-dominated profiles—reflects a chemical signature of escalating biological stress accumulating in the rhizosphere under intensifying intraspecific competition.

KEGG pathway enrichment analysis (*p* < 0.05) identified eight significantly enriched metabolic pathways, including ABC transporters; Galactose metabolism; Arachidonic acid metabolism; Cutin, suberine and wax biosynthesis; Biotin metabolism; Nucleotide metabolism; Tropane, piperidine and pyridine alkaloid biosynthesis; and Tryptophan metabolism (Figure 7b). To further identify core regulatory nodes in the metabolic network, we performed a KEGG topological analysis (Figure 7c), which jointly considers pathway enrichment significance (*p*-value) and pathway influence within the metabolic network (Impact value). Integrating statistical significance with metabolic network topology (*p* < 0.05 and pathway impact > 0.1) narrowed these down to three core pathways: Biotin metabolism; Tropane, piperidine, and Pyridine alkaloid biosynthesis; and Pantothenate and CoA biosynthesis (Figure 7c). Because certain pathways (such as Pantothenate and CoA biosynthesis) have high Impact values, they were identified as key pathways in the topological analysis, even though they did not reach statistical significance in the conventional enrichment analysis (Figure 7b). This reflects the differences in screening strategies between the two analytical methods. Within these pathways, four metabolites emerged as core contributors (VIP > 1, FDR < 0.1): N-Feruloyloctopamine, N-Carboxyethyl-Gamma-Aminobutyric Acid, Glutarate Semialdehyde, and Sutherlandin D.

Spearman correlation analysis (Figure 7d) revealed that all four core metabolites were broadly and significantly negatively correlated with seedling growth and photosynthetic performance. N-Feruloyloctopamine, N-Carboxyethyl-Gamma-Aminobutyric Acid, and Glutarate Semialdehyde showed significant negative correlations with all measured traits, including leaf area, taproot length, root and shoot biomass, *A*, *E*, *G*_s_, and *C*_i_. Sutherlandin D exhibited a similar but slightly attenuated pattern, with a non-significant association with plant height. Collectively, these findings indicate that high-density competition not only depletes the rhizosphere of growth-promoting metabolites but is also associated with the accumulation of bioactive compounds with potential autotoxic or allelopathic properties, potentially establishing a chemical legacy in the soil that may reinforce the growth suppression of *S. rostratum* under crowded conditions.

### 2.5. Multi-Omics Integration Reveals That the Root Exudate–Microbe–Metabolite Cascade Is Associated with Rhizosphere Remodeling Under Intraspecific Competition

**Figure 8:**
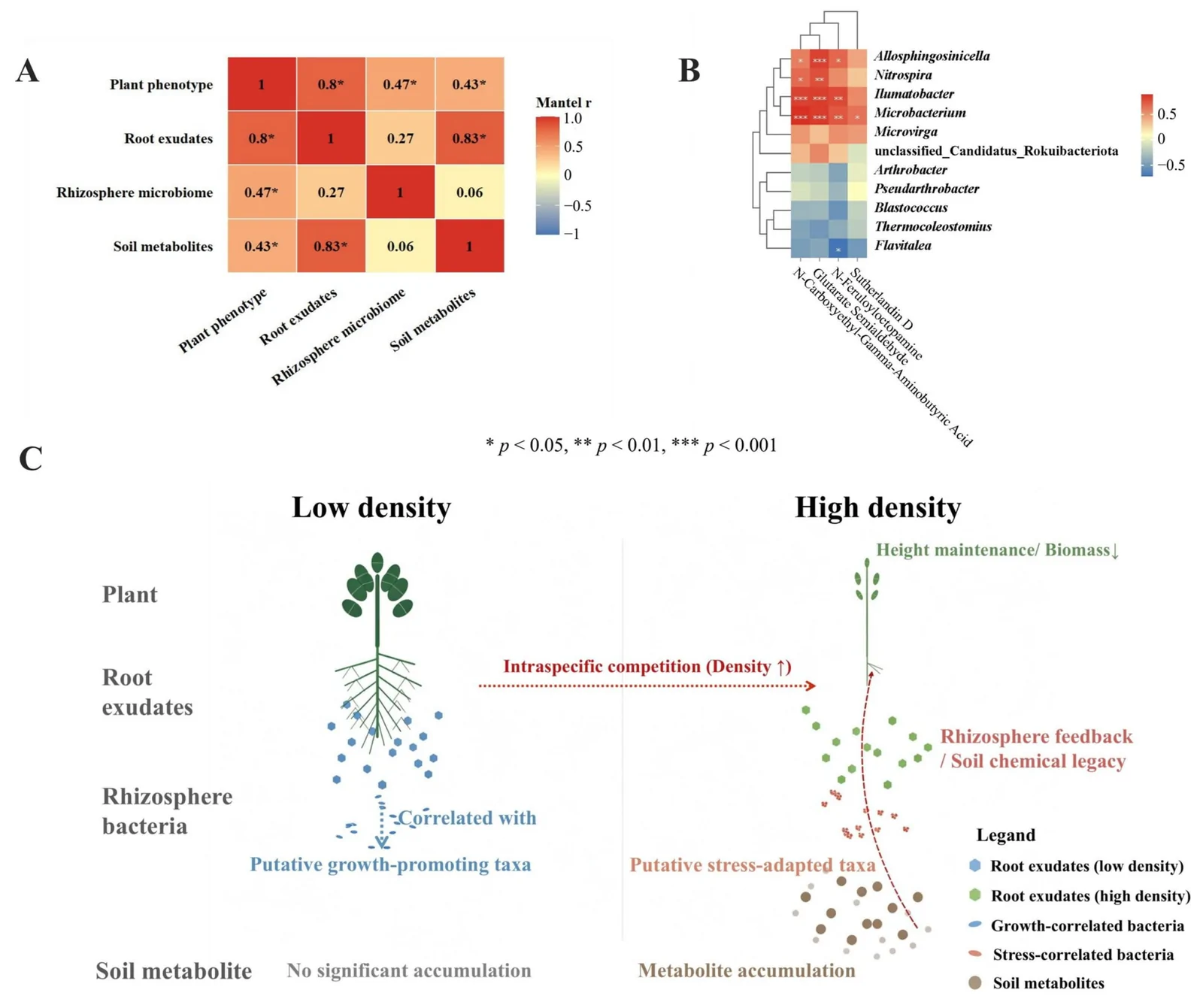
Multi-omics integration reveals that the root exudate–microbe–metabolite cascade is associated with rhizosphere remodeling under intraspecific competition. (**A**) Mantel test heatmap showing overall associations among plant phenotypes, root exudates, rhizosphere microorganisms, and soil metabolites. Numbers indicate Mantel *r*; asterisks indicate significance (* *p* < 0.05, ** *p* < 0.01, *** *p* < 0.001). (**B**) Spearman correlation heatmap of 11 density-responsive bacterial genera and 4 key soil metabolites. Red indicates positive correlation and blue indicates negative correlation; asterisks indicate significance after FDR correction (* *p* < 0.05, ** *p* < 0.01, *** *p* < 0.001). (**C**) A correlation-based conceptual model illustrating the density-dependent shifts in the rhizosphere. Under low density, root exudates are associated with putatively growth-promoting taxa. Under high density, shifts in root exudates are correlated with putatively stress-adapted taxa and the accumulation of soil metabolites. The horizontal red arrow indicates the direction of density increase, and the downward arrows indicate correlative links. Note: All dashed arrows indicate correlation or hypothesized relationships derived from multi-omics association analysis, rather than direct causal effects. The potential rhizosphere feedback loop requires further experimental validation.

To dissect how root exudates, the rhizosphere bacterial community, and soil metabolites jointly associate with rhizosphere remodeling under intraspecific competition, we performed the following analyses: (i) we integrated four layers of datasets (plant phenotypes, root exudates, rhizosphere bacterial community, and soil metabolites); (ii) we used Mantel tests to assess the associations among the four layers of data at the overall community level; (iii) we used variable-level Spearman correlation analysis to resolve specific pairwise relationships; and (iv) we constructed a conceptual model to summarize the cascade pathways associated with intraspecific competition (Figure 8).

The distance matrix-based Mantel test results showed (Figure 8A) that plant phenotypes were strongly correlated with root exudates (*r* = 0.80, *p* < 0.05) and root exudates were also highly correlated with soil metabolites (*r* = 0.83, *p* < 0.05). The associations of plant phenotypes with rhizosphere microorganisms and soil metabolites were moderate (*r* = 0.47 and 0.43, respectively, *p* < 0.05), and the association between root exudates and rhizosphere microorganisms was also significant but relatively weak (*r* = 0.27, *p* < 0.05). The microbial community and soil metabolites showed no significant correlation at the overall community level (*r* = 0.06, *p* > 0.05). This weak association may have resulted from dilution of the overall community distance matrix by numerous stable, non-responsive taxa; therefore, we further resolved the pairwise relationships between specific taxa and metabolites through variable-level correlation analysis.

The associations between root exudates and rhizosphere microorganisms are shown in Figure 5e; building on this, this section further focuses on the pairwise relationships between microorganisms and soil metabolites. Variable-level Spearman correlation analysis showed that density-responsive bacterial genera could be divided into two groups with opposite correlation patterns: putatively growth-associated taxa represented by *Flavitalea* and *Blastococcus*, which were positively correlated with plant biomass, and potentially stress-adapted taxa represented by *Allosphingosinicella*, *Ilumatobacter*, and *Microbacterium*, which were positively correlated with Glutarate Semialdehyde and Sutherlandin D (Figure 8B).

Integrating the above multi-omics evidence, we summarized a correlation-based cascade pathway of rhizosphere remodeling associated with intraspecific competition (Figure 8C): intensified intraspecific competition was associated with plant phenotypic remodeling and root exudate reprogramming; changes in exudate composition were associated with a shift in the rhizosphere bacterial community from putatively growth-associated taxa to putatively stress-adapted taxa; this microbial community reorganization was further associated with the accumulation of two growth-decline-related soil metabolites (Glutarate Semialdehyde and Sutherlandin D), potentially forming soil chemical legacies and potentially exerting feedback on subsequent seedling growth. The above exudate–microbe–metabolite association pattern provides multi-omics evidence for understanding rhizosphere remodeling in *S. rostratum* under intraspecific competition.

## 3. Discussion

### 3.1. Effects of Intraspecific Competition on Seedling Phenotype and Root Exudates

The effects of intraspecific competition on *S. rostratum* seedlings exhibited pronounced asymmetry. As intraspecific competition intensified, root architecture was significantly simplified, and biomass and photosynthetic rate declined substantially, whereas plant height showed only a slight increase followed by a decrease (Figure 1 and Figure 2). This pattern indicates that under resource-limited conditions, plants preferentially maintain aboveground light-competitive ability while reducing carbon investment in roots. Similar phenomena have been reported in other invasive plants [14], and recent work has shown that intraspecific competition significantly alters root exudate metabolic profiles [2]. Plant density can also reshape rhizosphere chemistry [3], and density-associated exudate remodeling may further exacerbate negative plant–soil feedback [4].

Phenotypic changes altered the plant’s carbon allocation pattern, most directly manifested as marked changes in root exudate composition. With the increase in intraspecific competition, exudates shifted from being dominated by phenolic acids to being dominated by sugars, peptides, and organic acids, showing a clear gradient along the competition intensity gradient (Figure 3). Mantel tests showed a strong correlation between plant phenotypes and exudates (*r* = 0.80, *p* < 0.05; Figure 8A), indicating that the two occurred synchronously. Root exudates are not passive products of photosynthetic carbon allocation but rather an active chemical strategy by which plants respond to environmental changes [15,16]. When intraspecific competition intensifies, plants alter the chemical composition of exudates to modify the rhizosphere microenvironment, providing chemical signals for subsequent microbial community responses [17].

Such changes in exudates may have functional adaptive significance. The decrease in phenolic acids and the increase in sugars and organic acids imply a shift from defensive exudation to resource-acquisition and stress-adaptation exudation [18]. This shift is consistent with a strategy of reallocating carbon resources and preferentially maintaining aboveground growth under intraspecific competition. Overall, these results support a potential linkage among intraspecific competition, plant phenotype, and root exudate composition, and provide a basis for understanding the chemical adaptation of invasive plants in high-density populations.

### 3.2. Exudate Changes and Rhizosphere Microbial Reassembly

Although Mantel tests showed only a weak correlation between root exudates and rhizosphere microorganisms at the overall community level (*r* = 0.27, *p* < 0.05; Figure 8A), variable-level correlation analysis revealed clear pairwise relationships. Phenolic acid exudates dominant under weak intraspecific competition (e.g., 2-Benzylsuccinic Acid) were positively correlated with genera such as *Flavitalea* and *Blastococcus*, whereas sugar, peptide, and organic acid exudates enriched under strong intraspecific competition (e.g., Citric Acid, Arg-Met-Ser) were positively correlated with genera such as *Allosphingosinicella*, *Ilumatobacter*, and *Microbacterium* (Figure 5e). These pairwise patterns indicate that as intraspecific competition intensifies, the rhizosphere bacterial community does not change randomly but instead shows a trend of shifting from putatively growth-associated taxa to potentially stress-adapted taxa.

The weak correlation at the overall community level is likely because the distance matrix was diluted by numerous stable, non-responsive taxa. When focusing on specific taxa, the associations between exudates and microorganisms became clear. This is consistent with recent studies: the chemical composition of root exudates can act as a selection pressure affecting substrate utilization and colonization ability of rhizosphere microorganisms [17]. A chemical–trophic filtering hypothesis also emphasizes that plant metabolites and soil nutrients jointly shape rhizosphere microbial assembly [19], and root metabolites are considered metabolic architects of beneficial microbiome assembly [20]. Methodologically, Mantel tests have been widely used to link rhizosphere microbiome and root exudate metabolome datasets [21]. For example, Cotton et al. found that benzoxazinoids secreted by maize roots directly regulate the structure of the rhizosphere microbiome [22]; Jiang et al. also demonstrated in an intercropping system that specific rhizosphere metabolites were associated with changes in the microbial community [23]. Feng et al. further pointed out that plants can reprogram the functional expression of growth-promoting bacteria through temporal changes in exudates [17].

Based on this evidence, we speculate that when intraspecific competition intensifies, the shift in exudates from phenolic acids to sugars, peptides, and organic acids may alter the rhizosphere chemical environment, thereby selectively affecting the growth and colonization of different microbial taxa; putatively growth-associated bacteria (e.g., *Flavitalea*) may rely more on phenolic acid substrates, whereas potentially stress-adapted bacteria (e.g., *Allosphingosinicella*) are more adapted to a rhizosphere environment dominated by sugars and organic acids. This chemical selection process may be an important factor associated with the microbial community shift from putatively growth-associated taxa to potentially stress-adapted taxa. More importantly, as functionally evidenced in Figure 6, the enrichment of pathways related to environmental sensing and transport (e.g., Two-component system and ABC transporters) provides direct functional support for this shift towards putatively stress-adapted taxa under intensified competition.

### 3.3. Microbial Reassembly and Soil Metabolite Accumulation

Mantel tests showed that the microbial community and soil metabolites were not significantly correlated at the overall community level (*r* = 0.06, *p* > 0.05; Figure 8A), but variable-level analysis revealed clear pairwise relationships. *Allosphingosinicella*, *Ilumatobacter*, and *Microbacterium*, enriched under strong intraspecific competition, were significantly positively correlated with Glutarate Semialdehyde and Sutherlandin D, whereas putatively growth-associated *Flavitalea* and *Blastococcus* were significantly negatively correlated with these two metabolites (Figure 8B). Meanwhile, these two metabolites were significantly negatively correlated with plant biomass, root traits, and photosynthetic parameters (Figure 7d). These results indicate that microbial community reassembly and the accumulation of specific soil metabolites occurred synchronously.

The weak correlation at the overall community level is likely due to dilution of the distance matrix by numerous non-responsive taxa, similar to the exudate–microbe relationship in Section 3.2. When focusing on specific taxa, the associations between microorganisms and metabolites became apparent. Similar phenomena have been reported in other systems. Korenblum et al. found that the rhizosphere microbiome can alter root metabolite secretion patterns through systemic signals [24]; Wang et al. confirmed in *Lithocarpus litseifolius* that genera such as *Burkholderia* were significantly positively correlated with the accumulation of dihydrochalcones in rhizosphere soil [25]; Baker et al. also identified key metabolites linking the rhizosphere microbiome and metabolome [26]. These studies all support an important role of microorganisms in rhizosphere metabolite accumulation. The soil metabolome is also considered a key link in plant–soil feedbacks [27], and root exudate metabolites can influence plant–soil feedbacks by shaping the rhizosphere microbiota [28]. In *S. rostratum*, Zhang et al. revealed linked responses among rhizosphere microbial communities, metabolite composition, and soil physicochemical properties from a comparison of invaded sites [29]. The present study, starting from an intraspecific competition gradient, further dissected the correlation-based “exudate–microbe–metabolite” cascade, providing evidence at a different level for understanding rhizosphere remodeling in this species.

Based on this, we propose a testable hypothesis: potentially stress-adapted bacteria enriched under intensified intraspecific competition may promote the accumulation of Glutarate Semialdehyde and Sutherlandin D in soil through metabolic transformation or interactions with plants, and these metabolites may further feedback to inhibit subsequent seedling growth. It should be noted that these inferences are based on correlation analysis and cannot establish causality; this hypothesis still needs to be verified through exogenous metabolite addition and inoculation experiments with key strains. In *S. rostratum*, previous studies have shown that it has allelopathic potential [11,30], and Liu et al. further found that intraspecific allelopathic effects are regulated by interactions between litter and soil microorganisms [12]. This study extends this framework to a living root system, revealing the association chain from exudates to microbial reassembly to metabolite accumulation under intraspecific competition.

### 3.4. Implications for Intraspecific Competition Strategies of Invasive Plants

Invasive plants often form high-density monodominant populations in novel habitats; intraspecific competition therefore becomes an important regulatory factor in their population dynamics. Traditional views hold that resource depletion and autotoxic effects caused by high density limit population expansion, but this study shows that *S. rostratum* does not passively endure density stress under intraspecific competition; instead, it buffers this stress through multilevel rhizosphere remodeling. It should be noted that in natural high-density populations, density effects are inherently coupled with a reduction in per-plant resource availability [31]. It is undeniable that the fixed soil volume in our pot experiment means that high density inevitably leads to reduced per-plant resource availability. In this context, our study did not aim to isolate resource limitation from competitive signaling but rather to evaluate the integrated ecological consequences of density stress for the rhizosphere microecosystem. The observed shift from phenolic acids to sugar/peptide exudates, and the subsequent microbial reassembly, represent the plant’s active strategy to cope with this combined stress (e.g., by recruiting stress-adapted microbes to enhance nutrient acquisition or stress tolerance), rather than a passive result of nutrient depletion alone.

This study observed that when intraspecific competition intensified, plants preferentially maintained plant height. We speculate that this maintenance of height might be a strategy to compete for light, although shading experiments are required to disentangle above- and belowground competition in future studies, while root exudates shifted from phenolic acids to sugars, peptides, and organic acids. These exudates were associated with the enrichment of specific microbial taxa and further occurred synchronously with the accumulation of potentially inhibitory metabolites. This “exudate–microbe–metabolite” cascade pathway may help plants maintain relative stability of rhizosphere function in high-density populations. Recent studies also support this view: Tang et al. found that the diversity and stability of the rhizosphere microbial community of *S. rostratum* were significantly higher than those of its native congeners [32], and Liu et al. demonstrated that litter input and soil microbial communities regulate the intraspecific allelopathic effects of *S. rostratum* [12]. These findings corroborate the rhizosphere remodeling strategy proposed in this study. Rhizosphere microbes can influence intraspecific competition in the invasive plant *Ageratina adenophora* [5], and invasive plant competitiveness may be jointly determined by nitrogen use strategies and the rhizosphere microbiome [6]. Soil biota can also shift the competitive ability of invasive plants [33], and intraspecific variation in indirect plant–soil feedbacks can influence wetland plant invasion [13].

This rhizosphere remodeling strategy offers a potential explanation for the ‘density paradox’ in invasion ecology: how invasive plants sustain high population growth at high densities. While classical hypotheses, such as ‘enemy release’ and ‘biotic resistance’, primarily focus on the absence of specialized natural enemies in newly invaded ranges, they largely overlook the plant’s own capacity to actively modulate its rhizosphere microenvironment to buffer against intraspecific competition. This study suggests that invasive plants may construct rhizosphere conditions favorable to themselves, or at least not detrimental to their own population establishment, under high density through exudate-associated microbial reassembly and soil chemical legacies. Similarly, Wang et al. found that *Mikania micrantha* reshapes rhizosphere nitrogen cycling through microbial recruitment and allelopathic regulation [10], and Evans et al. also confirmed that intraspecific competition in the invasive plant *Alliaria petiolata* can contribute to soil-mediated ecological–evolutionary feedback [34]. These cases, together with this study, point to a trend: under high-density intraspecific competition, invasive plants may achieve adaptive regulation through rhizosphere chemical and microbial pathways.

Of course, this study is based on correlation analysis and cannot establish causality. Whether this cascade pathway is general and its actual role in field multispecies communities still require further verification through exogenous addition, strain inoculation, and field density gradient experiments. However, it is clear that the rhizosphere remodeling ability exhibited by *S. rostratum* under intraspecific competition provides a possible mechanistic explanation for its formation of high-density populations in invaded ranges and offers new ideas for density-dependent management of invasive plants.

### 3.5. Limitations and Future Directions

Several limitations should be acknowledged. First, the destructive pulling and 24-h hydroponic collection may induce wounding stress and microbial degradation. Future studies should employ non-destructive in-situ collection methods (e.g., absorbent resin traps or root windows) or sterile hydroponic systems. Second, our fixed-soil-volume pot design cannot disentangle competitive signaling from resource limitation, so the observed rhizosphere effects should be interpreted as an integrated response to both. Future studies using split-root systems or continuous nutrient replenishment are needed to decouple these mechanisms. Third, the annotation rate of non-targeted metabolomics was limited. The identified metabolites should be considered putative identifications and require validation. Fourth, the small sample size (*n* = 3) increases the risk of overfitting. Finally, although we identified specific functional shifts at KEGG Pathway Level 3, our analysis was limited to pathway-level abundances. Deeper exploration, such as specific gene-level annotations, was not extensively performed. Future studies are needed to integrate functional metagenomics to substantiate these ecological labels. Furthermore, it must be acknowledged that this study is primarily based on correlation analysis and cannot establish direct causality. The proposed cascade pathway is a conceptual model; future studies involving exogenous metabolite addition and strain inoculation are required to verify these causal relationships.

## 4. Materials and Methods

### 4.1. Plant Material and Soil Preparation

To accurately assess the impact of plant invasion on native soil ecosystems, the experimental soil was collected from uninvaded patches within a localized area generally invaded by *S. rostratum* in Shiren Gou, Shuimogou District, Urumqi, Xinjiang, China (43°49′ N, 87°46′ E, elevation c. 950 m). We specifically selected sampling microsites completely free from *S. rostratum* growth to represent the pristine, pre-invasion baseline soil conditions. After removing stones and plant debris, the soil was air-dried, passed through a 2-mm sieve, thoroughly homogenized, and used unsterilized to preserve the native soil microbial community. *S. rostratum* seeds were collected from the same region. To break seed dormancy and ensure uniform germination, seeds were surface-sterilized with 3% sodium hypochlorite for 10 min, rinsed with sterile distilled water, treated with 75% ethanol for 1–2 min, and rinsed thoroughly again. The sterilized seeds were then soaked in a 400 mg L^−1^ gibberellic acid (GA_3_) solution and incubated at 30 °C for 24 h before being air-dried on filter paper [35,36].

### 4.2. Experimental Design and Pot Setup

The treatments were set as 1 (One), 3 (Three), 5 (Five), and 10 (Ten) plants per pot. In order to investigate the impact of plant invasion on the soil of primitive habitats, we also set up a blank control group without plant growth (CK). Each treatment comprised three biological replicates, totaling 15 pots. Uniform plastic pots (top diameter 20 cm, bottom diameter 15 cm, height 18 cm; surface area c. 0.03 m^2^) were filled with 500 g of the prepared experimental soil. The soil was initially watered to approximately 60% of its field capacity. Pre-treated seeds were uniformly sown into the pots. Upon emergence and the development of 2–3 true leaves, seedlings were thinned from reserve pots to achieve the exact target densities. All pots were maintained in a controlled growth chamber under a 12 h light/12 h dark photoperiod, at 30 °C/20 °C (day/night), with a constant light intensity of 350 μmol m^−2^ s^−1^. During the cultivation period, 30 mL deionized water was added every 3 days. Pot positions were randomized weekly to avoid microenvironmental edge effects. The experiment was terminated at the end of the vegetative growth stage for uniform harvesting and sampling.

### 4.3. Plant Growth and Photosynthetic Physiological Measurements

One day prior to harvest, photosynthetic gas exchange parameters were measured between 09:00 and 11:00. Three fully expanded, mature leaves from each treatment were randomly selected to record net photosynthetic rate (*A*), transpiration rate (*E*), stomatal conductance (*G*_s_), and intercellular CO_2_ concentration (*C*_i_) using a portable photosynthesis system (LI-6800, LI-COR Inc., Lincoln, NE, USA). Measurement conditions were set at a light intensity of 350 μmol m^−2^ s^−1^ and a reference CO_2_ concentration of 400 μmol mol^−1^.

At harvest, whole plants were carefully extracted, and roots were gently washed with low-pressure running water to remove adhering soil. Plant height and maximum taproot length were recorded. Leaf area was determined using a smart leaf area scanner (YMJ-CHA3, Top Cloud-agriculture Technology Co., Ltd., Hangzhou, China). Plants were then partitioned into shoots and roots, and fresh weights were immediately recorded. Intact roots were scanned using a high-resolution root scanner, and morphological parameters (total root length, surface area, volume, and number of root tips) were analyzed using WinRHIZO Pro 2024a software (Regent Instruments Inc., Quebec, QC, Canada). Finally, tissues were oven-dried at 105 °C for 30 min to deactivate enzymes, followed by drying at 80 °C to constant mass to determine dry weight.

To account for the varying number of plants per pot across different density treatments, growth and physiological parameters were measured for all individual plants within each pot, and the values were averaged at the pot level for all statistical analyses. Thus, each pot served as an independent biological replicate (*n* = 3).

### 4.4. Root Exudate Collection and Non-Targeted Metabolomics

Root exudates were collected hydroponically according to Shao et al. [30] with minor modifications.

Intact *S. rostratum* seedlings at the late vegetative stage were carefully uprooted with minimal mechanical disturbance, and roots were gently rinsed with sterile deionized water. The entire root system was submerged in a glass beaker containing 200 mL of sterile deionized water. Beakers were wrapped in aluminum foil to prevent light exposure to the roots. Following 24 h in the growth chamber, the collection solution was filtered through a 0.22 μm sterile membrane and snap-frozen at −80 °C. The frozen samples were lyophilized and then sent to Majorbio Bio-Pharm Technology Co., Ltd. (Shanghai, China), for LC-MS/MS non-targeted metabolomic analysis. To minimize the confounding effects of mechanical wounding, all seedlings were handled with identical gentle procedures across all treatments. The collection time (24 h) and temperature were strictly controlled to standardize the background level of potential microbial degradation. No antimicrobial agent was added during the collection period to avoid chemical interference, and the potential microbial degradation of exudates was acknowledged as a limitation. The extraction, chromatography, and mass spectrometry procedures were performed following the service provider’s standard protocols. To standardize the exudate data for the different number of plants per pot, the raw metabolite intensities of the Three, Five, and Ten treatments were normalized (divided) by the respective number of plants (3, 5, and 10) to obtain the single-plant equivalent exudate concentration prior to downstream statistical analyses.

Metabolomic profiling and raw data processing were performed following Salem et al. [37]. Root exudate samples were subjected to non-targeted LC-MS/MS analysis. Metabolite annotation, data preprocessing, and multivariate statistical analyses (PCA and OPLS-DA) were performed following standard protocols. Detailed procedures are provided in Supplementary Methods S1.

### 4.5. Rhizosphere Soil Sampling and Multi-Omics Analysis

Harvesting procedures were conducted under aseptic conditions. After shaking off loose bulk soil, the soil tightly adhering to the roots (0–2 mm) was collected with a sterile brush and designated as rhizosphere soil. For the CK treatment, soil was mixed evenly and then sampled from the pot. Soil samples were divided into two aliquots and flash-frozen in liquid nitrogen for metagenomic and metabolomic analyses.

Soil metagenomic sequencing and analysis. Metagenomic DNA was extracted, and shotgun metagenomic sequencing was performed on the Illumina NovaSeq platform (Illumina, San Diego, CA, USA). After quality control and assembly, the raw sequences were aligned against the NR and KEGG databases for taxonomic and functional annotation. Differentially abundant microbial groups were identified through alpha/beta diversity analysis, ANOSIM/Adonis tests, and LEfSe analysis. Detailed procedures are provided in Supplementary Method S2.

Soil metabolomic analysis. Soil metabolites were extracted and subjected to LC-MS/MS analysis following the same protocol used for root exudates. KEGG pathway enrichment analysis was then performed. Detailed procedures are provided in Supplementary Methods S1.

### 4.6. Statistical Integration and Analysis

Data were organized using Excel 2021(Microsoft Corp., Redmond, WA, USA), and statistical analyses were performed using SPSS 25.0 (IBM Corp., Armonk, NY, USA). Multiple group comparisons were conducted using one-way ANOVA followed by Tukey–Kramer post-hoc tests (*p* < 0.05). Morphological and physiological figures were generated using OriginPro 2021 (OriginLab Corp., Northampton, MA, USA), and omics analysis figures were obtained from the Majorbio Cloud Platform.

Differential root exudates were screened based on the PLS-DA model using the criteria VIP > 0.7, |log_2_FC| > 0.6, and FDR < 0.05. Root exudate metabolite intensities were normalized by the number of plants per pot (i.e., divided by 3, 5, and 10) to account for the pooled collection of roots and to obtain a single-plant equivalent concentration. Differential rhizosphere bacterial genera were screened based on LEfSe analysis (LDA > 3, *p* < 0.05). Key soil metabolites were screened based on the OPLS-DA model (VIP > 1, FDR < 0.1). Multi-omics integration adopted a two-step strategy. Before constructing Euclidean distance matrices, all phenotypic, root exudate, and soil metabolite data were z-score-standardized to eliminate scale effects. First, distance matrices were constructed based on Euclidean distance (plant phenotypes, root exudates, and soil metabolites) and Bray–Curtis distance (rhizosphere bacterial community), and Mantel tests were used to assess associations among the four layers of data at the overall community level, with 999 permutations [21]. Second, for density-responsive variables, Spearman rank correlation analysis was used to resolve variable-level pairwise relationships (root exudates × microorganisms, microorganisms × soil metabolites), with multiple testing correction using the Benjamini–Hochberg method (FDR < 0.05).

Given the limited biological replication (*n* = 3), the PLS-DA and OPLS-DA models were primarily used for exploratory visualization. To evaluate model robustness, permutation tests were performed, and the models were interpreted cautiously in the context of biological relevance rather than strict predictive power.

## 5. Conclusions

This study integrated four layers of data, including plant phenotypes, root exudates, the rhizosphere bacterial community, and soil metabolites, to reveal the rhizosphere remodeling strategy of *S. rostratum* under intraspecific competition. The effects of intraspecific competition on seedlings were asymmetric: root architecture was simplified, and biomass and photosynthetic rate decreased significantly, whereas plant height showed only a slight increase followed by a decrease, indicating that plants preferentially maintain aboveground light-competitive ability. As intraspecific competition intensified, root exudates shifted from being dominated by phenolic acids to being dominated by sugars, peptides, and organic acids, and changes in exudate composition occurred synchronously with rhizosphere microbial reassembly: phenolic acid exudates dominant under weak competition were positively correlated with putatively growth-associated bacteria such as *Flavitalea* and *Blastococcus*, whereas sugar/peptide exudates enriched under strong competition were positively correlated with potentially stress-adapted bacteria such as *Allosphingosinicella*, *Ilumatobacter*, and *Microbacterium*. Microbial reassembly was further associated with the accumulation of Glutarate Semialdehyde and Sutherlandin D in soil; these two metabolites were significantly negatively correlated with plant biomass, root traits, and photosynthetic parameters and may constitute a soil chemical legacy that exerts potential feedback on subsequent seedling growth. In summary, *S. rostratum* remodels the rhizosphere through an “exudate–microbe–metabolite” cascade under intraspecific competition, and this strategy may help it tolerate high-density population stress and partly explain its invasion success. Because this study is based on correlation analysis, the causal relationships of the above cascade pathway still require further verification through exogenous addition and strain inoculation experiments.

## Data Availability

The raw data supporting the conclusions of this article will be made available by the authors upon request.

## Supplementary Materials

The following supporting information can be downloaded at: https://www.mdpi.com/article/10.3390/plants15213439/s1, Figure S1: Contiguous growth of *S. rostratum* in invaded areas; Figure S2: Root morphology scans; Figure S3: PLS-DA of root exudates; Figure S4: Volcano plots and Venn diagram; Figure S5: UpSet plot; Figure S6: KEGG Pathway Level 2 functional heatmap; Figure S7: CAZy functional heatmap at the Class level; Figure S8: PLS-DA of soil metabolites; Table S1: Root morphological parameters; Table S2: Cohen’s *d* effect sizes; Table S3: Relative abundance of dominant bacterial genera; Method S1: Metabolomics data processing; Method S2: Metagenomic sequencing and analysis.
