# Supplementary material for: Density-Dependent Changes in Root Exudates, Rhizosphere Microbes, and Soil Metabolites of Solanum rostratum
Source: Plants (Basel). 2026 Nov 5;15(21):3439. doi: 10.3390/plants15213439 (PMC13765553; doi:10.3390/plants15213439)
Supplement: Supplementary file 1 [file plants-15-03439-s001.zip › plants-4637575-supplementary.pdf]

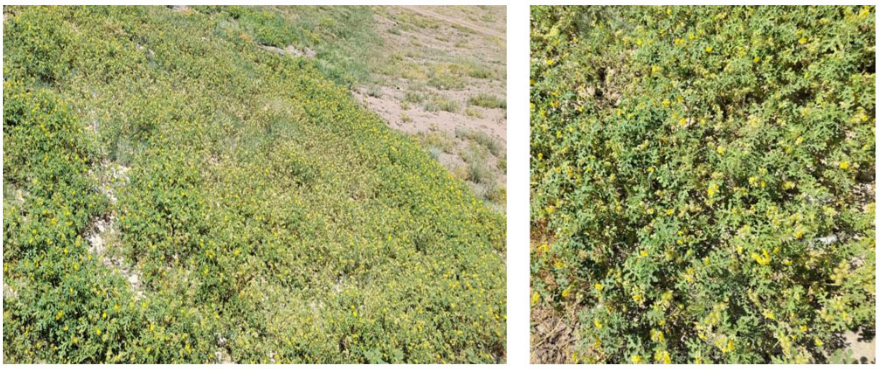

Figure S1.Contiguous growth of *S. rostratum* in invaded areas. Photograph showing high-density monodominant patches of *S. rostratum* in the invaded habitat.

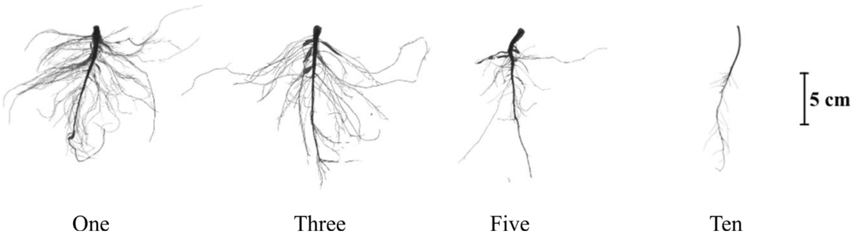

Figure S2. Root morphology scans of *S. rostratum* seedlings under different plant densities. Roots were scanned at the end of the vegetative growth stage. From left to right: One, Three, Five, and Ten plants per pot. Scale bar = 5 cm.

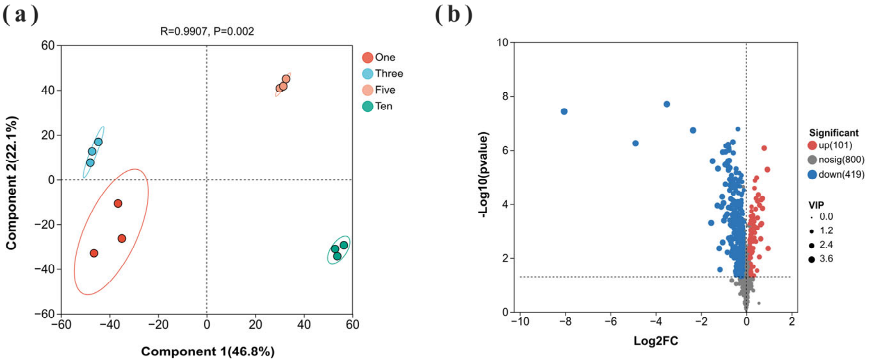

Figure S3. PLS-DA analysis of root exudates from *S. rostratum* under different plant densities and screening of differential root exudates. (a) PLS-DA score plot showing clear separation among the One, Three, Five, and Ten groups. (b) Volcano plot of differential root exudates between the One and Ten groups. Red dots indicate significantly differential exudates (VIP > 0.7, $|\log_2 FC| > 0.6$, FDR < 0.05); grey dots indicate non-significant ones. The x-axis represents $\log_2$ fold change, and the y-axis represents $-\log_{10}$(FDR).

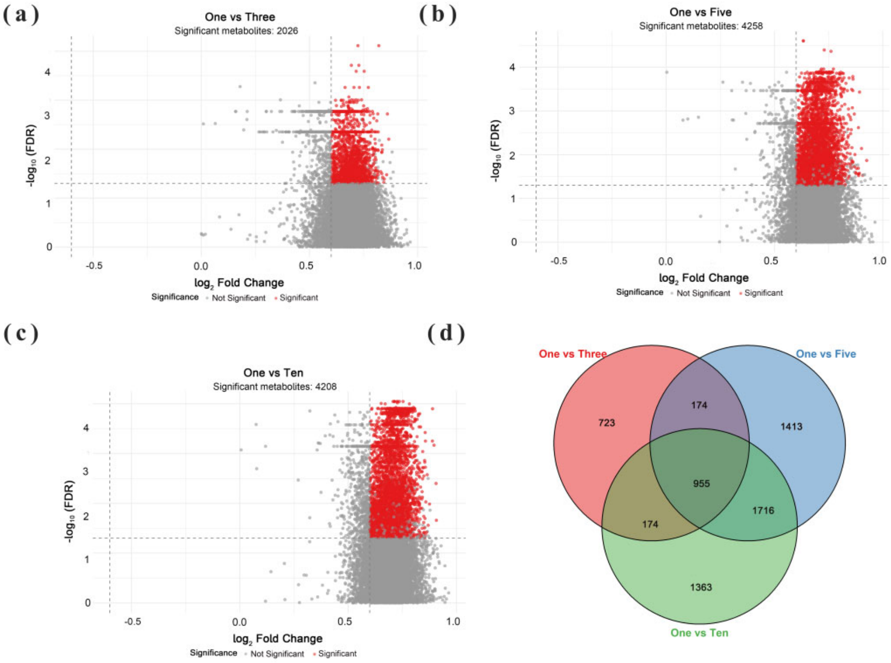

Figure S4. Volcano plots and Venn diagram of differential root exudates. (a – c) Volcano plots for the three comparison groups: (a) One vs. Three, (b) One vs. Five, (c) One vs. Ten. Red dots indicate significantly differential exudates, grey dots indicate non-significant ones. (d) The intersection contains 955 shared differential metabolites, of which 13 were confidently annotated against the HMDB/Metlin databases and retained for downstream analysis.

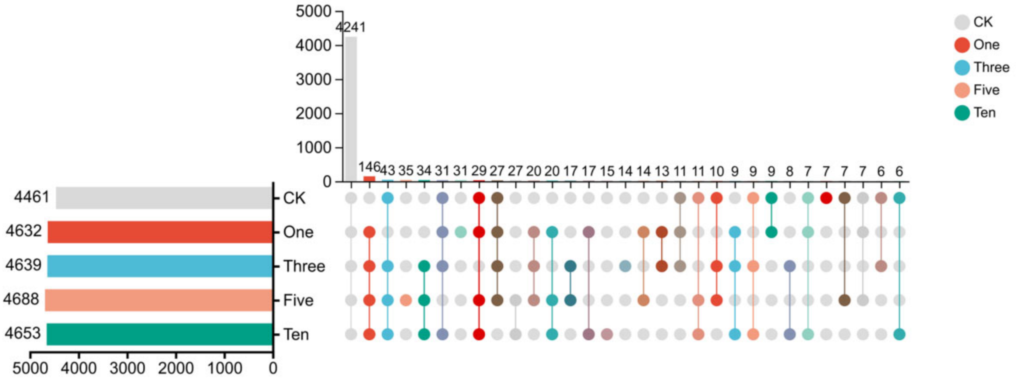

Figure S5. UpSet plot illustrating the shared and unique rhizosphere bacterial genera at the genus level under different planting densities. The horizontal bar chart on the left shows the total number of genera in each group (CK, One, Three, Five, Ten); the vertical bar chart on top represents the size of the intersections (shared genera) corresponding to specific group combinations; the dot-and-line matrix indicates which groups are involved in each intersection. The tallest bar on the far left represents the number of genera shared by all five groups (4,241 genera).

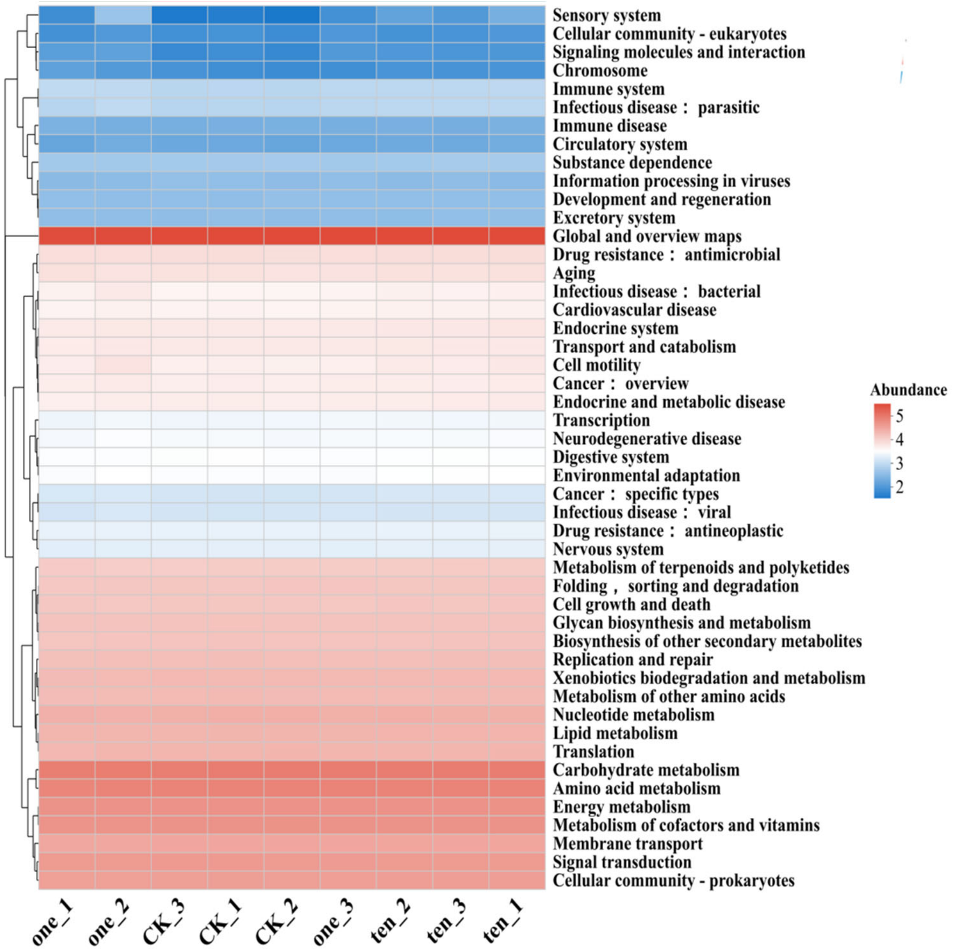

Figure S6. KEGG Pathway Level 2 functional heatmap. The heatmap displays the relative abundances of major functional categories across CK, One, and Ten treatments. The relatively stable color patterns across different density groups indicate high functional redundancy at the macroscopic functional level within the rhizosphere microbiome.

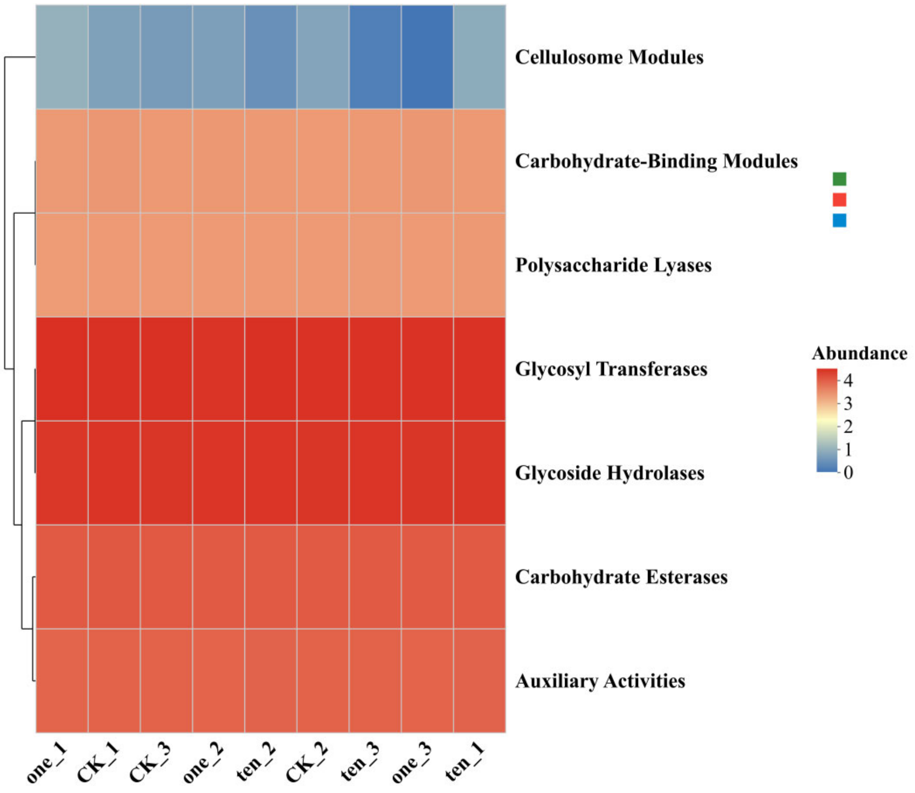

Figure S7. CAZy functional heatmap at the Class level. The heatmap shows the relative abundances of carbohydrate-active enzyme classes (log_{10}-transformed TPM values) across CK, One, and Ten treatments. Core enzyme classes, such as Glycoside Hydrolases (GHs) and Glycosyl Transferases (GTs), remained relatively stable across densities, further supporting the presence of functional redundancy in carbohydrate utilization.

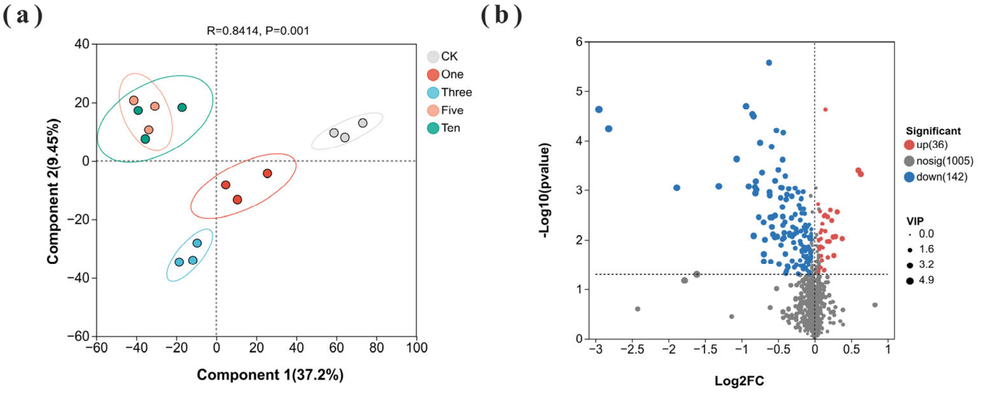

Figure S8. PLS-DA analysis of soil metabolites from *S. rostratum* under different plant densities and screening of differential metabolites. (a) PLS-DA score plot showing treatment-dependent separation of soil metabolite profiles. (b) Volcano plot of differential soil metabolites between the One and Ten groups. Red dots indicate significantly up-regulated metabolites; blue dots indicate significantly down-regulated metabolites; grey dots indicate non-significant ones.

Table S1. Root morphological parameters of *S. rostratum* seedlings under different plant densities. Data represent the combined results of root scans for all seedlings in each pot. Treatments: One, Three, Five, and Ten plants per pot.

Treatment	Total root length (cm)	Projected area (cm^2)	Surface area (cm^2)	Volume (cm^3)	Number of root tips
One	446.25	12.99	40.83	0.974	893
Three	221.40	6.84	21.49	0.449	320
Five	75.09	2.62	8.22	0.182	117
Ten	36.79	1.15	3.61	0.057	61

Table S2. Cohen's *d* effect sizes for growth and physiological traits (One vs. Ten). Effect magnitude was classified as very large ($|d| > 3.7$). Traits include leaf area, taproot length, root fresh weight, root dry weight, plant height, shoot fresh weight, shoot dry weight, E, A, C_i, and G_s.

Trait	Mean One	Mean Ten	SD pooled	Cohen's *d*	Effect magnitude
Leaf area	3687.82	777.00	126.98	22.92	Very large
Taproot length	9.04	5.82	0.27	11.99	Very large
Root fresh weight	84.44	16.00	5.45	12.56	Very large
Root dry weight	23.89	4.10	0.73	27.29	Very large
Plant height	6.07	5.20	0.17	5.26	Very large
Shoot fresh weight	1101.36	181.10	22.07	41.70	Very large
Shoot dry weight	177.33	38.57	7.61	18.24	Very large
E	5.41	1.70	0.20	18.85	Very large
A	10.34	7.48	0.76	3.76	Very large
C_i	309.54	215.55	15.57	6.04	Very large
G_s	188.09	82.92	8.31	12.66	Very large

Table S3. Relative abundance and significance of differences of dominant bacterial genera in rhizosphere soil under different planting density treatments. Data are presented as mean ± standard deviation (n = 3 per group). Treatment groups: CK (unplanted control), One, Three, Five, Ten. P values are based on the Kruskal–Wallis rank-sum tests.

Genus	CK	One	Three	Five	Ten	***P*-value**
Allosphingosinicella	8875.85 ± 709.69	7596.43 ± 1416.10	8938.38 ± 400.53	10459.09 ± 1342.48	12977.20 ± 1300.98	0.042
Arthrobacter	13418.29 ± 1149.44	25503.99 ± 5670.90	31700.28 ± 1736.21	27664.01 ± 3652.24	16423.60 ± 1851.43	0.019

Blastococcus	29127.30 ± 1002.68	31814.56 ± 2576.00	32633.61 ± 1010.59	31979.31 ± 1752.71	26668.77 ± 1313.47	0.037
Flavitalea	4774.77 ± 236.00	7297.86 ± 1707.30	6390.70 ± 636.88	5782.90 ± 754.52	5225.25 ± 300.82	0.027
Ilumatobacter	6652.24 ± 112.54	7209.14 ± 369.80	7822.43 ± 439.59	8145.72 ± 477.59	9594.89 ± 914.44	0.015
Microbacterium	4069.07 ± 226.52	4198.31 ± 246.20	5325.56 ± 1007.81	4862.94 ± 232.15	8582.38 ± 4331.88	0.034
Microvirga	22950.89 ± 191.88	23042.72 ± 1170.00	26901.12 ± 622.79	25986.99 ± 1770.83	26742.79 ± 318.03	0.043
Nitrospira	20650.81 ± 1027.57	14342.34 ± 787.70	16025.25 ± 520.36	15174.98 ± 185.02	16569.86 ± 534.51	0.011
Pseudarthrobacter	1493.56 ± 145.74	3295.58 ± 826.30	4674.64 ± 384.09	4009.86 ± 715.18	2104.72 ± 255.65	0.013
Thermocoleostomius	16.39 ± 1.53	3803.48 ± 6038.02	279.71 ± 210.00	8505.21 ± 14430.96	111.92 ± 124.18	0.045
Unclassified_Candidatus_Rokuibacteriota	22800.45 ± 1040.05	19504.01 ± 1699.50	20651.21 ± 451.62	19707.71 ± 604.73	21582.31 ± 396.05	0.02

Method S1. Metabolomics data processing and analysis. Raw data were processed using Progenesis QI for peak detection, alignment, and integration. Metabolite annotation was conducted against the HMDB, Metlin, and an in-house database. Data preprocessing involved removing features with >20% missing values per group, imputing remaining missing values with the minimum value, applying total sum normalization, and excluding variables with relative standard deviation (RSD) > 30% in quality control (QC) samples. The final $\log_{10}$-transformed data matrix was analyzed using PCA and OPLS-DA via the R package ropls. For differential metabolite screening, the intersection of each comparison group was obtained through volcano plot and Venn diagram analyses, and only metabolites that could be unambiguously annotated against HMDB and Metlin databases were retained for downstream analysis..The confidence level of metabolite identification was evaluated based on MSI standards. In this study, metabolites were putatively annotated (MSI Level 2 or 3), and further verification with authentic standards was not performed.

Method S2. Metagenomic sequencing and analysis. Total DNA was extracted from 1–3 g of rhizosphere soil using the E.Z.N.A. Soil DNA Kit (Omega Bio-tek, Norcross, GA, USA). DNA quality was assessed using a NanoDrop 2000 and Qubit 4.0. Shotgun metagenomic sequencing was performed on the Illumina NovaSeq platform. Clean reads, filtered by fastp (v0.20.0), were assembled using MEGAHIT (v1.2.9). Open reading frames (ORFs) were predicted via Prodigal (v2.6.3), and a non-redundant gene catalog was constructed using CD-HIT (v4.6.1). Taxonomic and functional annotations were obtained by aligning amino acid sequences against the NR and KEGG databases using Diamond (e-value ≤ $1e^{-5}$). Alpha and beta diversity (PCA based on Bray–Curtis distance), ANOSIM/Adonis tests, and LEfSe analysis (LDA > 3, $P < 0.05$) were performed to identify significantly distinct microbial taxa.
